# Exploring patient engagement in atrial fibrillation with multimorbidity: impact on quality of life, medication adherence and healthcare perceptions—a multicountry cross-sectional study

**DOI:** 10.1136/bmjopen-2024-094351

**Published:** 2025-03-18

**Authors:** Caterina Bosio, Dilara Usta, Donato Leo, Caterina Trevisan, Deirdre Lane, Guendalina Graffigna, AFFIRMO Project Consortium, Søren Påske Johnsen

**Affiliations:** 1 EngageMinds HUB – Consumer, Food and Health Engagement Research Center, Universita Cattolica del Sacro Cuore, Cremona, Lombardia, Italy; 2 Department of Cardiovascular and Metabolic Medicine, University of Liverpool, Liverpool, UK; 3 Liverpool Centre for Cardiovascular Science, Liverpool Heart and Chest Hospital NHS Trust, Liverpool, Liverpool, UK; 4 Department of Medicine, Università degli Studi di Padova, Padova, Veneto, Italy; 5 Department of Medical Sciences, University of Ferrara Department of Medical Sciences, Ferrara, Emilia-Romagna, Italy; 6 Aging Research Center, Karolinska Institutet, Stockholm, Stockholm, Sweden; 7 Liverpool Centre for Cardiovascular Science, Liverpool Heart and Chest Hospital NHS Foundation Trust, Liverpool, Liverpool, UK; 8 Department of Clinical Medicine, Aalborg University, Aalborg, Region Nordjylland, Denmark

**Keywords:** Medication Adherence, Multimorbidity, Quality of Life, Patient-Centered Care, Patient Reported Outcome Measures

## Abstract

**Abstract:**

**Objective:**

To examine patient engagement (PE) levels of atrial fibrillation (AF) patients with multimorbidity, to identify distinct personas based on sociodemographic and clinical characteristics, as well as engagement levels, and to compare PE in disease management with health-related quality of life, medication adherence, and perceptions of care quality.

**Design:**

A cross-sectional survey.

**Setting:**

Data were collected through an online survey platform between 31 May 2022 and 31 January 2023 from five European countries (Denmark, Italy, Romania, Spain and the UK).

**Participants:**

The study involved 659 AF patients older than 18 years who were diagnosed with one or more concomitant chronic health conditions.

**Primary and secondary outcome measures:**

The survey focused on identifying the needs and quality performance indicators (QPIs) of patients. Emotional engagement was evaluated using the Patient Health Engagement Scale (PHE-s), and cognitive-behavioural engagement was assessed using the Altarum Consumer Engagement Measure (ACE). Engagement scores of each measure were grouped as high or low and compared by age group, sex, level of education and country of recruitment, health-related quality of life, medication adherence and perception of care quality using χ^2^ and Mann‒Whitney U tests (p<0.05).

**Results:**

Among the 659 AF patients (70.9±10.2 years, 52.8% female), 428 (65%) were categorised as having high emotional PE levels based on PHE-s and were significantly more likely to be <75 years old and male, have a secondary level of education or above, and have <3 comorbidities (p<0.05). Regarding the ACE scores, 369 (56%) were classified as having high cognitive-behavioural PE levels and were more likely to be <65 years old, reside in Northern Europe, have degree-level education or higher, and have <3 comorbidities (p<0.05). Additionally, participants with high emotional PE demonstrated better quality of life, medication adherence and perceptions of quality of care, whereas those with higher levels of cognitive-behavioural PE had better quality of life and perceptions of quality of care.

**Conclusions:**

From a clinical perspective, the findings highlight the need for a personalised approach sensitive to the expectations and needs of AF patients. The present research suggests that implementing sociodemographic and clinical profiling for AF patients could facilitate the formulation of improved care strategies.

**STRENGTHS AND LIMITATIONS OF THIS STUDY**

- This study offers a comprehensive analysis of patient engagement in atrial fibrillation (AF) management, using validated tools (PHE-s and ACE) to assess both emotional and cognitive-behavioural dimensions.
- It explores a wide range of sociodemographic and clinical factors, offering a nuanced understanding of their impact on patient engagement in AF management, also addressing quality of life, medication adherence and perceptions of quality of care.
- The sample, predominantly European with under-representation from specific countries, may limit the findings’ applicability to more diverse global populations.
- Despite rigorous translation and validation efforts, variations in cultural and linguistic contexts may have influenced the comparability of survey responses.

## Introduction

 Atrial fibrillation (AF) is the most prevalent type of sustained cardiac arrhythmia globally,^1^ with projections indicating an increasing prevalence due to ageing populations and an increasing incidence of predisposing risk factors such as hypertension, obesity and diabetes mellitus.^2^ AF not only exerts a substantial burden on healthcare systems but also significantly impacts patients’ quality of life^3^ while increasing the risk of stroke, heart failure and mortality.^4^ Patients with AF require long-term management involving rate and rhythm control, anticoagulation therapy and prevention of complications.^1^

In recent years, a notable paradigm shift has occurred in managing AF, with a growing emphasis on patient engagement and involvement in decision-making processes.^5 6^ Traditional approaches to AF management predominantly focus on stroke prophylaxis and rhythm and rate control strategies, often overlooking crucial aspects of patient preferences, values and goals.^7^ However, the advent of patient-centred care models has underscored the necessity of integrating patient perspectives into treatment strategies and promoting patient engagement to improve clinical outcomes, enhance patient satisfaction and optimise the efficiency of healthcare delivery.^8^ From this perspective, numerous studies have reported the advantages of promoting patient engagement in managing AF.^9^,^11^ Patient engagement is defined as patients actively participating in activities or decisions that impact the patient community, leveraging their specific knowledge and relevant experiences as patients.^12^ A 6-month follow-up study conducted with 136 AF patients demonstrated that smart digital tools to support patient education and engagement significantly improved medication adherence.^9^ Another study involving two cohorts of AF patients (406 patients before the stroke event and 518 patients at discharge from the hospital) reported that patients with AF exhibit better adherence to anticoagulation therapy, resulting in reduced stroke and systemic embolism rates.^10^ Similarly, an observational descriptive real-life study revealed that patients with AF who actively participated in shared decision-making processes regarding rhythm control strategies experienced improved treatment satisfaction and adherence and reported better health-related quality of life.^11^ The evidence suggests that promoting patient engagement in AF management holds immense promise for improving clinical outcomes, enhancing patient satisfaction and optimising the efficiency of healthcare delivery.

Despite the growing recognition of the importance of patient engagement in AF management, significant challenges still persist in translating these principles into clinical practice.^13^ Healthcare providers often face barriers such as time constraints, resource limitations and insufficient training in patient-centred communication skills.^5^ In addition, patients encounter obstacles such as low health literacy, cultural disparities and limited access to healthcare services, which impede their ability to actively engage in care.^5 14 15^ For this reason, efforts to integrate patient perspectives into treatment strategies are pivotal for delivering patient-centred care and achieving superior outcomes in AF management.^16^ Although evidence suggests that high levels of patient engagement underpin better healthcare journeys,^17^ systematic clinical assessments of patient engagement levels remain rare,^18^ and this issue is further compounded in AF care, impeding the practical achievement of a truly personalised approach to promoting patient engagement throughout the patient journey.

Therefore, the European Union Horizon 2020 research programme has funded the ‘Atrial fibrillation integrated approach in frail, multimorbid and polymedicated older people’ (AFFIRMO) Program (Grant Agreement no. 899871). Patient engagement was planned as a key pillar of the AFFIRMO project, which is dedicated to developing a multidisciplinary and integrated digital approach to treating multimorbid AF patients. On the basis of these premises, the current study aims to explore patient engagement and evaluate whether patients’ level of engagement is related to different experiences and needs in their disease management: health-related quality of life, medication adherence and their perceptions of quality of care.

## Methods

### Study design and participants

A cross-sectional study was conducted with a convenience sample of patients with AF from five partner countries of the AFFIRMO project (the UK, Italy, Spain, Denmark and Romania) from 31 May 2022 to 31 January 2023. Patients with AF were eligible for inclusion if they met the following criteria: (1) aged ≥18 years and (2) the presence of at least one chronic comorbid condition. Exclusion criteria included the following: (1) inability to provide informed consent, (2) moderate or severe cognitive impairment (eg, dementia), (3) inability to complete the survey online, (4) the presence of health conditions that impede survey completion, and (5) unwillingness to participate. Patients were invited to participate in the online survey through announcements on the Atrial Fibrillation Association (AFA) website or via healthcare professionals, including cardiologists, general practitioners, geriatricians, haematologists and internal medicine specialists. These professionals were contacted via email through professional networks within the project consortium and invited to share the survey with patients attending clinical appointments at participating hospitals.

### Instruments

The online platform hosting the survey for data collection was developed by the project partners, and the survey content was generated by academic partners and patient organisations. Before the data collection, all the questionnaires were translated from English to Italian, Romanian, Spanish and Danish, and the respective country leaders approved the translated versions of the online survey. The survey focused on identifying the needs and quality performance indicators (QPIs) of patients with AF and multimorbidity and included questionnaires to assess health-related quality of life, perceptions of quality of healthcare, medication adherence and patient engagement. The survey also recorded data on sociodemographic and clinical-related characteristics to better identify needs and QPIs for specific categories of patients. Details on the development of the survey and the questionnaires have been reported previously.^19^

#### Sociodemographics and health-related characteristics

The sociodemographic data included participants’ age, sex, ethnicity, country, educational attainment, current employment status, marital status, living arrangements, need for assistance and smoking habits. The participants’ self-reported health-related data included their existing comorbidities (in terms of the type and severity of disease) and whether they had been hospitalised in the previous year.

#### Health-related quality of life

Health-related quality of life was assessed with the European Quality of Life Survey-5 Dimension-3 Levels (EQ-5D-3L) instrument,^20^ which comprises five dimensions related to (1) mobility, (2) self-care, (3) usual activities, (4) pain/discomfort, and (5) anxiety/depression. Each of these items can be scored by ticking one of the three-level options available: (1) no problems, (2) some problems, or (3) extreme problems. Additionally, the European Quality visual analogue scale (VAS) included in the questionnaire allows patients to score their health based on their perceptions. The final scores vary from 0 (worst health) to 100 (best health), and a higher score indicates a good health-related quality of life on the EQ-5D-3L.

#### Perceptions of quality of healthcare

The Healthcare Climate Questionnaire (HCCQ) was used to assess patients’ perceptions of the degree to which their specific doctor or team of healthcare providers is autonomous and supportive.^21^ The short version of the survey involves six items on a 7-point Likert systems scale ranging from 1 (strongly disagree) to 7 (strongly agree) and is scored by averaging the individual item scores. The scores range from 6 to 42, where a higher HCCQ score indicates a higher perception of the quality of healthcare. Sample items include ‘I feel that my doctor has provided me choices and options’, ‘I feel understood by my doctor’ and ‘My doctor encourages me to ask questions.’ Compared with the initial 15-item scale, which demonstrated strong internal consistency (Cronbach’s α=0.95) and a single-factor structure,^22^ the present study also yielded high internal consistency (Cronbach’s α=0.96) and a unifactorial arrangement.

#### Medication adherence

Medication adherence was evaluated with the Medication Adherence Report Scale (MARS-5),^23^ a validated assessment tool for measuring patients’ non-adherence to medication. The MARS-5 questionnaire consists of five aspects of non-adherence behaviour: forgetting, changing dosage, stopping, skipping and taking less medication, with the following response scales: ‘always, often, sometimes, rarely, and never’. Sample items include ‘I forget to take them’, ‘I stop taking them for a while’ and ‘I decide to skip a dose.’ On MARS-5, one item evaluates unintentional non-adherence, while four items evaluate intentional non-adherence. The sum scores range between 5 and 25 points, where a higher MARS-5 score indicates greater self-reported medication adherence.

#### Patient engagement

The survey employed two patient engagement questionnaires to evaluate the two distinct dimensions of the patient experience: (1) the Patient Health Engagement Scale (PHE-s) for emotional engagement and (2) the Altarum Consumer Engagement Measure (ACE measure) for cognitive–behavioural engagement.

The PHE-s is a validated tool designed for patients to assess their engagement in healthcare based on the Patient Health Engagement Model; it describes patient engagement within the psychosocial needs of individuals and introduces four engagement positions: ‘blackout, arousal, adhesion, and eudaimonic project’.^24^ The instrument involves seven responses with a single factor and ordinal structure, also enabling patients to place themselves in intermediate positions and avoid social desirability bias. The sample items of the scale include ‘When I think about my illness, I feel overwhelmed by emotions’, ‘I feel anxious every time a new symptom arises’, ‘I am used to my illness’ and ‘I find my life meaningful despite my illness’. In the current study, the PHE-s scores were dichotomised into low and high categories (cut-off PHE-s: scores <3 and ≥3, respectively). The original scale demonstrated good internal reliability (ordinal α=0.85), and the present study demonstrated high internal consistency (Cronbach’s α=0.86).

The ACE measure was developed to assess the health engagement of individuals and populations from multiple aspects of patient perceptions, participation in health and healthcare activities, and the use of information to compare and choose providers or services.^25^ The tool was originally developed with 21 items, but it was subsequently shortened to 12 items consisting of three subscales: commitment (four items), informed choice (four items) and navigation (four items). The ACE-12 uses a 5-point response scale: strongly disagree, somewhat disagree, neither agree nor disagree, somewhat agree and strongly agree, and higher scores represent greater patient engagement. The sample items of the instrument are as follows: ‘I can handle my health well’, ‘When I work to improve my health, I succeed’ and ‘I have brought my own information about my health to show my doctor.’ The ACE measure levels were dichotomised into low and high categories (cut-off ACE: bottom 50% and top 50%, respectively). The psychometric properties of the ACE-12 showed acceptable internal consistency (commitment subscale ordinal α=0.750, informed choice subscale ordinal α=0.710 and navigation subscale ordinal α=0.54).^26^ The current study yielded acceptable internal consistency (Cronbach’s α=0.73).

### Data analysis

Statistical analyses were conducted via IBM SPSS V.29 (IBM Corp., Armonk, New York, USA). The categorical variables are represented as frequencies (percentages), and the continuous variables are presented as the means (SD) or medians ± IQRs, as appropriate, for descriptive data. The normality of the data was assessed via the Shapiro‒Wilk test, which yielded a p value <0.001, indicating that the data were not normally distributed. To assess whether the study had sufficient statistical power to detect differences between high and low patient engagement groups, a post hoc power analysis was conducted using G* Power V.3.1.9.7.^27^ Consequently, non-parametric tests were employed for subsequent data analysis. Using a medium effect size assumption (r=0.3)^28^ and the significance level of α=0.05, the power was computed using the Mann-Whitney U test to compare high and low patient engagement groups. For the PHE-s engagement groups (n=428 vs n=231), the achieved power was 94.7%, while for the ACE measure engagement groups (n=369 vs n=290), the achieved power was 96.1%. Since both values exceed the conventional 80% threshold, the present study had sufficient power to detect true differences between engagement groups. Additionally, the Mann‒Whitney U test was used to examine sex-related differences in questionnaire scores, whereas the Kruskal‒Wallis test was used to assess the variations in questionnaire scores across different age groups. Following these tests, a post hoc analysis was conducted to discern differences between groups, employing pairwise comparisons with significance values adjusted via the Bonferroni correction method. A p value <0.05 was considered statistically significant.

A partial correlation analysis explored correlations (p<0.05) between PHE-s and ACE measure scores while controlling for age and sex. Pearson’s correlation coefficients were employed to assess the relationships among variables. The characteristics of engagement personas were subsequently evaluated by categorising PHE-s and ACE measure scores to quantify the engagement level of patients. These personas were then analysed and stratified by age, sex, level of education, country of recruitment and number of comorbidities. Differences between the low-engagement and high-engagement groups were assessed via a χ^2^ test, and pairwise comparisons were conducted via a Z-score test with Bonferroni correction. The effect of age on engagement level (high and low groups) was evaluated via the Mann‒Whitney U test. Owing to the limited recruitment of patients in Denmark (n=3) and to enable cross-country comparisons, Danish patients were grouped with patients from the UK. Comparative analyses were performed between patients from Northern Europe (the UK and Denmark), Eastern Europe (Romania) and Southern Europe (Spain and Italy).

### Patient and public involvement statement

Patients and/or the public were not involved in the design, conduct, reporting or dissemination plans of this research.

## Results

### Participants’ sociodemographic and health-related characteristics

A total of 659 AF patients completed the survey. The average age was 70.9 (10.2) years, 52.8% were female, 97.9% were White and most participants (54.3%) were recruited from the UK. In addition, a majority had completed a secondary level of education (39.9%) and had a degree level or above (42.5%), and approximately half of them (55.1%) had three or more comorbidities. An overview of the sample characteristics is presented in online supplemental appendix A.

### Differences in patient engagement by sex, age, level of education and country

Patient engagement assessed by the PHE-s revealed a significant difference between sexes, with men reporting greater health engagement than women. However, no discernible differences were observed across various age groups (see table 1). In contrast, the ACE measure highlighted significant differences in healthcare engagement between younger adults (aged <65 years) and those aged ≥65 years, with the former demonstrating notably higher levels of engagement in healthcare decisions (see table 1). Interestingly, no significant differences were detected based on sex within the age groups assessed.

**Table 1:** Differences in patient engagement levels by sex, age, country, educational level and number of comorbidities (n=659)

Variables/patient engagement levels	Patient engagement (PHE-s)	Patient engagement (ACE measure)
Sex		
Overall	3.0 (2.0–3.0)	52.0 (46.7–58.7)
Male	3.0 (2.0–3.0)**	52.4 (46.7–58.0)
Female	3.0 (2.0–3.0)	52.8 (46.7–58.7)
P value**†**	<0.001*	0.207
Age groups		
18–64 years	3.0 (2.0–3.0)	54.6 (49.3–61.3)‡
65–74 years	3.0 (2.0–3.0)	52.0 (45.3–57.3)
75+ years	3.0 (2.0–3.0)	52.0 (46.6–57.3)
P value†	0.294	<0.001*
Countries		
Overall	3.0 (2.0–3.0)	52.0 (46.7–58.7)
Eastern Europe	3.0 (2.0–4.0)	53.3 (46.7–59.7)
Northern Europe	3.0 (2.0–3.0)	53.3 (48.0–58.7)
Southern Europe	3.0 (2.0–3.0)§	50.7 (45.3–57.3)
P value†	0.031*	0.030*
Educational attainment		
Overall	3.0 (2.0–3.0)	52.0 (46.7–58.7)
Primary school	3.0 (2.0–3.0)	49.3 (42.7–54.7)
Secondary school¶	3.0 (2.0–3.0)	52.0 (46.7–58.7)
Degree level or above	3.0 (2.0–3.0)	53.3 (48.0–58.7)***
P value†	0.053	<0.001*
Number of comorbidities		
≤2	3.0 (2.0–3.0)††	53.3 (49.3–58.7)‡‡
3–5	3.0 (2.0–3.0)	52.0 (46.7–57.3)
>5	3.0 (2.0–3.0)	49.3 (44.0–57.3)
P value†	<0.001*	<0.001*

*Statistically significant.

* *Significantly greater than that of women.

† Kruskal‒Wallis test was performed.

‡ Significantly greater than that in the 18–64 years age group.

§ Significantly higher than that in Northern Europe.

¶ High school and Apprentice/Professional Training/Vocational Training were included in the secondary school education group.

** *Significantly higher than those in the primary school education group.

†† Significantly greater than that of the group with 3–5 comorbidities.

‡‡ Significantly greater than in the groups with 3–5 and >5 comorbidities.

ACE measureAltarum Consumer Engagement MeasurePHE-sPatient Health Engagement Scale

Preliminary analysis revealed apparent differences in patient engagement across regions, as indicated by both the PHE and ACE measure scores (see table 1). However, subsequent pairwise comparisons, adjusted via the Bonferroni correction method, failed to detect any statistically significant differences between the groups. There was a significant difference in patient engagement based on the level of education but only for the ACE measure. Specifically, patients with a degree-level education or higher exhibited greater engagement with healthcare than patients with lower education levels did. However, there were no differences in patient engagement based on educational level when assessed by the PHE-s (see table 1).

### Impact of comorbidities on patient engagement level

Patient engagement in healthcare demonstrated significant differences depending on the number of comorbidities, as indicated by both the PHE-s (χ^2^=11.893, p=0.003) and the ACE measures (χ^2^=15.473, p=<0.0001) (see table 1). Specifically, individuals with fewer than three comorbidities reported higher levels of engagement in managing their healthcare than did patients with three or more comorbidities (see table 1).

### Engagement personas

**Figure 1:**
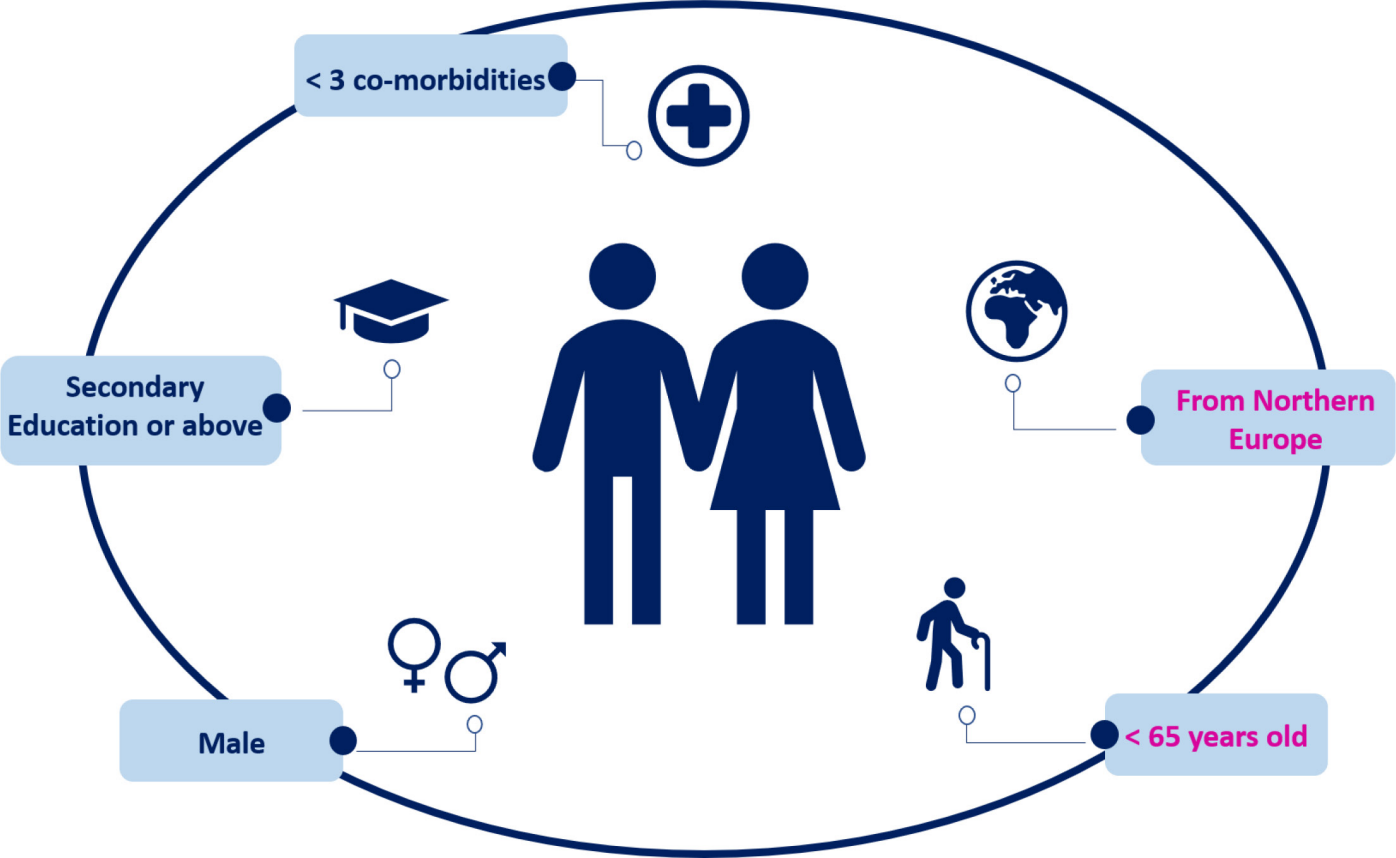
Characteristics of patients with a high engagement persona. The blue text indicates significant results from the PHE-s, and the pink text indicates significant results from the ACE measure.

Using data derived from the PHE-s and ACE measures, the characteristics of patient engagement personas were delineated (see figure 1). According to the PHE-s, 428 (65%) individuals were classified as exhibiting ‘high’ engagement, whereas 231 (35%) individuals were categorised as demonstrating ‘low’ engagement. Patients in the high emotional engagement group were younger than 75 years of age (χ²=6.457, p=0.040), were more likely to be male (χ²=15.425, p<0.0001), possessed a secondary level of education or above (χ²=9.028, p=0.029) and had fewer than three comorbidities (χ²=11.893, p=0.004) than individuals in the low emotional engagement group were. No significant differences in high versus low engagement were observed based on the country of recruitment (see table 2).

**Table 2:** Engagement personas: participants were grouped by high and low levels of engagement according to their PHE-s and ACE scores (n=659)

	Patient engagement level(PHE-s)			Patient engagement level(ACE measure)		
	High(n=428)(65%)	Low(n=231)(35%)	P value	High(n=369)(56%)	Low(n=290)(44%)	P value
Age groups (*years*)						
<65	96 (14.6)	60 (9.1)		105 (15.9)†	51 (7.7)	
65–74	144 (21.9)	93 (14.1)	0.040*	122 (19.1)	111 (17.4)	0.005*
≥75	188 (28.5)	78 (11.8)‡		123 (17.3)	114 (18.4)	
Male sex	226 (34.3)†	85 (12.9)	< 0.001*	165 (25.0)	146 (22.2)	0.151
Country						
Eastern Europe	58 (8.8)	34 (5.2)		54 (8.2)	38 (5.8)	
Northern Europe	230 (34.9)	131 (19.9)	0.546	215 (32.6)†	146 (22.2)	0.034*
Southern Europe	140 (21.2)	66 (10.0)		100 (15.2)	106 (16.1)‡	
Educational attainment (n=640)						
Primary	62 (9.7)	32 (5.0)		38 (5.9)	56 (8.8)‡	
Secondary§	156 (24.4)†	110 (17.2)	0.029*	141 (22.0)	125 (19.5)	<0.001*
Degree level or above	195 (30.5)†	85 (13.3)		176 (27.5)†	104 (16.3)	
Number of comorbidities (n=638)						
≤2	198 (31.0)†	77 (12.1)		173 (26.3)†	102 (15.5)	
3–5	182 (28.5)	115 (18.0)	0.004*	156 (24.5)	141 (21.1)	<0.001*
>5	35 (5.5)	31 (4.9)		25 (3.9)	41 (6.4)‡	

* Statistically significant.

† Patient engagement is significantly greater than it is in the total sample.

‡ Patient engagement is significantly lower than that of the total sample.

§ High school and Apprentice/Professional Training/Vocational Training were included in the ‘Secondary level’ education group.

ACE measureAltarum Consumer Engagement MeasurePHE-sPatient Health Engagement Scale

Based on the ACE measure, 369 (56%) individuals were categorised as reporting ‘high’ engagement (median score ≥50), whereas 290 (44%) individuals were classified as demonstrating ‘low’ engagement. Patients in the high cognitive-behavioural engagement group were more likely to be younger than 65 years (χ²=10.680, p=0.005), reside in Northern Europe (χ²=6.773, p=0.034), have a degree-level education, or higher (χ²=17.975, p<0.0001), and have fewer than three comorbidities (χ²=15.473, p<0.0001) than those in the low cognitive-behavioural engagement group were (see table 2). However, no significant differences in sex distribution were observed between patients in the high and low cognitive-behavioural engagement groups (see table 2).

### Correlation analyses

A partial correlation analysis, controlling for sex and age, revealed a weak yet statistically significant positive correlation in patient engagement between the PHE-s and ACE measure scores (rs(655)=0.265, p<0.001). Mobility (r=−0.258, p<0.001), self-care (r=−0.199, p<0.001), usual activities (r=−0.299, p<0.001), pain/discomfort (r=−0.297, p<0.001) and anxiety/depression (r=−0.437, p<0.001) demonstrated significant negative relationships with the PHE-s scores. Conversely, the perception of overall quality of life (r=0.424, p<0.001), medication adherence (r=0.111, p<0.05) and the perception of quality of healthcare (r=0.260, p<0.001) exhibited significant positive correlations wi(th the PHE-s scores.

Furthermore, mobility (r=−0.209, p<0.001), self-care (r=−0.236, p<0.001), usual activities (r=−0.198, p<0.001), pain/discomfort (r=−0.122, p<0.05) and anxiety/depression (r=−0.167, p<0.05) were significantly and negatively associated with the ACE measure scores. In contrast, perceptions of overall quality of life (r=0.203, p<0.001), medication adherence (r=0.111, p<0.05) and quality of healthcare (r=0.245, p<0.001) demonstrated significant positive correlations with the ACE measure scores. The Pearson correlation variables among the study variables can be found in the supplementary material (online supplemental appendix B).

### Differences in quality of life, medication adherence and perception of quality of life by PHE-s and ACE measure scores

Differences in quality of life were observed based on patient engagement. AF patients with lower levels of emotional engagement, as assessed by the PHE-s, reported significantly greater impairments in mobility, self-care and usual activities, along with higher levels of pain/discomfort and anxiety/depression (p<0.001) (see table 3). Conversely, patients with higher levels of emotional engagement reported an overall better quality of life (VAS score 50.0 (45.0–65.0) vs 74.0 (60.0–85.0), p<0.001). Additionally, self-reported medication adherence (p=0.007) and perceptions of quality of care (p<0.001) were significantly greater among AF patients reporting greater levels of emotional engagement (see table 3).

**Table 3:** Differences in quality of life, medication adherence and perception of quality of life by PHE-s and ACE measure scores

Questionnaire(median (IQR))	Patient engagement level(PHE-s)			Patient engagement level(ACE measure)		
	High(n=428)	Low (n=231)	P value^**^	High(n=369)	Low (n=290)	P value^**^
Quality of life (EQ-5D-3L)						
Mobility	1.0 (1.0–2.0)	2.0 (1.0–2.0)†	<0.001*	1.0 (1.0–2.0)	2.0 (1.0–2.0)†	<0.001*
Self-care	1.0 (1.0–1.0)	1.0 (1.0–2.0)†	<0.001*	1.0 (1.0–1.0)	1.0 (1.0–2.0)†	<0.001*
Usual activities	1.0 (1.0–2.0)	2.0 (1.0–2.0)†	<0.001*	1.0 (1.0–2.0)	2.0 (1.0–2.0)†	<0.001*
Pain/discomfort	1.0 (1.0–2.0)	2.0 (1.0–2.0)†	<0.001*	2.0 (1.0–2.0)	2.0 (1.0–2.0)†	0.023*
Anxiety/depression	1.0 (1.0–2.0)	2.0 (2.0–2.0)†	<0.001*	1.0 (1.0–2.0)	2.0 (1.0–2.0)†	0.002*
VAS	74.0 (60.0–85.0)†	50.0 (45.0–65.0)	<0.001*	70.0 (50.0–80.0)†	60.0 (50.0–75.0)	<0.001*
Medication adherence (MARS-5)	24.0 (23.0–25.0)†	24.0 (22.0–25.0)	0.007*	24.0 (22.0–25.0)	24.0 (22.0–25.0)	0.224
Perception of quality of care (HCCQ)	5 (3.7–6.3)†	4.0 (2.7–5.8)	<0.001*	5.0 (3.7–6.3)†	4.2 (3.0–5.8)	<0.001*

*Statistically significant.

* *Mann‒Whitney U test was performed.

† Scale/subscale scores are significantly higher than those of the other group.

eVASvisual analogue scaleHCCQHealth Care Climate QuestionnaireMARS-5Medication Adherence Report ScalePHE-sPatient Health Engagement Scale

Similarly, AF patients with lower levels of cognitive-behavioural engagement, as assessed by the ACE measure, reported significantly greater impairments in mobility, self-care and usual activities (p<0.001), as well as higher levels of pain/discomfort and anxiety/depression (p=0.23 and p=0.002, respectively) (see table 3). Conversely, patients with higher levels of cognitive-behavioural engagement reported a better overall quality of life (VAS score 60.0 (50.0−75.0) vs 70.0 (50.0–80.0), p<0.001). While there were no differences in self-reported medication adherence based on cognitive-behavioural engagement levels, the perception of quality of care (p<0.001) was significantly greater among AF patients reporting greater levels of cognitive-behavioural engagement (see table 3).

## Discussion

The present study provides a comprehensive analysis of patient engagement among AF patients with multimorbidity, highlighting several critical sociodemographic and health-related factors, including quality of life, treatment adherence and perceived quality of healthcare, that influence health engagement levels. Our findings underscore significant differences in patient engagement based on age, sex, educational level and number of comorbidities while also delineating distinct engagement personas within the AF patient population. The variation in patient engagement personas stems from discrepancies in the theoretical context employed by each scale. The PHE-s measures engagement as the level of psychological readiness and emotional engagement,^24^ whereas the ACE measures three domains of cognitive-behavioural engagement (commitment to everyday health behaviours, informed choice and navigation).^24^

The differences in findings between the age groups support the multidimensional nature of patient engagement, particularly within the context of AF management. Specifically, individuals younger than 75 years demonstrated greater emotional engagement than those 75 years and older did, and those aged <65 years had greater cognitive-behavioural engagement than those aged 65 years and older did. The higher emotional engagement among relatively older adults (< 75 years), as assessed by the PHE-s, can be attributed to several factors. First, older patients might have accumulated more experience managing their health conditions over time, leading to a deeper emotional investment in their healthcare journey.^29^ This prolonged exposure often leads to greater psychological adjustment to the disease, enhancing individuals’ confidence and emotional investment in their health management strategies. The Patient Health Engagement Model suggests that as patients gain knowledge and experience with their disease, they become more emotionally adjusted and confident in managing it.^12^ This is particularly relevant for older adults who have likely spent more years navigating the healthcare system and dealing with chronic conditions such as AF.^30^ Their increased familiarity with their condition may foster a sense of empowerment and emotional resilience, resulting in higher engagement levels. The life stage of older adults might drive them to be more proactive in maintaining their health,^31^ as they recognise the importance of managing their condition to preserve their quality of life.^32^ Moreover, younger adults (<65 years) showed greater cognitive-behavioural engagement according to the ACE measure. One possible explanation is that younger adults are often more attuned to the necessity of preventive health measures and may have fewer comorbidities than older adults do, allowing them to focus more on active health management strategies.^33^ Additionally, younger adults are often more inclined to seek information, participate in healthcare decisions and implement lifestyle changes to manage their disease effectively.^34^ This demographic tends to be more health literate and may have fewer cognitive impairments, enabling them to understand and apply complex health information more effectively.^35^ Moreover, the cognitive-behavioural domain involves a high degree of self-regulation, planning and execution of health-related activities, such as regular physical activity, dietary modifications, anticoagulant therapy and the diligent use of health monitoring devices to track heart rhythms and other vital signs,^36^ which younger adults might be more adept at due to better cognitive function and fewer age-related impairments.

Atrial fibrillation can present with different symptoms in men and women, potentially influencing engagement levels. Our results revealed that men reported higher levels of emotional engagement than women did; this finding is noteworthy, as it contrasts with some previous studies, which suggest that women generally show greater engagement in managing chronic conditions because of their tendency to use healthcare services more frequently and engage in preventive behaviours.^37 38^ However, one possible explanation for this discrepancy in AF self-management could be the difference in symptom perception and reporting. The literature on AF management indicates that female patients often report more disease-specific symptoms, including palpitations, dyspnoea, fatigue and dizziness, than men do.^39^ A substantially greater symptom burden can lead to reduced motivation and capacity to engage in AF self-management in women, also causing emotional stress, resulting in the avoidance of healthcare and reluctance to actively participate in their care. Studies have also shown that women with AF experience more atypical symptoms, such as weakness and/or fatigue,^40 41^ which could lead to under-recognition and less proactive engagement in managing their condition. While women may experience greater symptom burden, men might interpret their symptoms differently or under-report them, leading to a perception of lower symptom severity. In contrast to emotional engagement, cognitive-behavioural engagement did not significantly differ according to sex. This finding suggests that while male patients may exhibit greater emotional engagement, regardless of sex, both male and female patients might demonstrate similar levels of proactive and informed actions in managing their health^42^ and engage in similar cognitive and behavioural strategies to manage their condition.

Across the countries included in our study, no significant differences were observed in emotional engagement levels, indicating a relatively consistent emotional engagement profile among AF patients across different regions. On the other hand, cognitive-behavioural engagement exhibited significant differences across countries, suggesting that patients from Northern Europe demonstrated higher levels of cognitive-behavioural engagement than those from other regions. Some factors may contribute to these differences in cognitive-behavioural engagement across countries. For example, variations in healthcare infrastructure and access to resources may play a significant role. Northern European countries are often at the forefront of implementing digital health solutions and promoting patient-centred care.^43^ The availability of advanced healthcare technologies, along with a strong emphasis on patient empowerment and shared decision-making,^44^ may contribute to higher levels of cognitive-behavioural engagement among AF patients in these regions.

Our analysis indicated that patients with a degree-level education or higher presented greater emotional and cognitive-behavioural engagement than those with lower education levels. Individuals with higher levels of education tend to possess greater health literacy and demonstrate better self-care practices, enabling them to understand better and navigate the complexities of their health condition.^45^,^47^ Moreover, higher education levels are often associated with increased self-efficacy,^48^ which refers to an individual’s belief in their ability to execute behaviours necessary to produce desired outcomes successfully.^49^ As a result, AF patients with higher educational attainment may feel more empowered and confident in managing their health.^50^ Furthermore, education serves as a proxy for socioeconomic status, which can influence access to healthcare services and resources. AF patients with higher education levels may have greater financial resources and access to quality healthcare, facilitating their engagement in proactive health behaviours.

Patients with fewer than three comorbidities reported higher levels of health engagement in managing their healthcare than patients with more comorbidities did, suggesting that a greater comorbidity burden may negatively impact emotional adjustment and cognitive-behavioural engagement in managing health among AF patients. The increased complexity and burden of managing multiple health conditions may lead to feeling overwhelmed^51^ and reduced confidence in one’s ability to manage one’s health effectively. The literature suggests that patients with multiple comorbidities are at greater risk of adverse health outcomes, greater symptom burden and non-adherence to treatment recommendations due to multiple conditions.^52^,^54^ Moreover, the presence of multiple comorbidities is often associated with greater symptom burden, polypharmacy, functional limitations and healthcare utilisation,^55^,^57^ which may further exacerbate feelings of distress, jeopardise emotional adjustment and impede AF patients’ ability to actively manage their condition in terms of participating in shared decision-making, adhering to treatment regimens and adopting healthy lifestyle behaviours.

Our findings underscore the significant and positive relationship between patient engagement and quality of life, indicating that AF patients with higher engagement levels demonstrated significantly better mobility, self-care and capacity to perform usual activities, alongside reduced pain/discomfort and lower anxiety/depression scores. These results are consistent with the literature, which posits that engaged patients are more likely to adhere to treatment regimens, adopt healthy behaviours, manage their conditions more effectively, report better health outcomes and improve their well-being and quality of life.^58 59^ This relationship is likely due to the multifaceted benefits of engagement, which include increased health knowledge, enhanced self-efficacy and greater psychological resilience.^46 60 61^ Research also indicates that patient engagement and adherence to specific care pathways, such as the atrial fibrillation better care (ABC) pathway, enhance the quality of life in clinically complex Chinese patients with AF and multimorbidity or polypharmacy.^62^ Furthermore, interventions aimed at supporting patient engagement have demonstrated positive effects on the quality of life of AF patients, underscoring the importance of holistic approaches in managing AF in the presence of multimorbidity.^63^

Our results confirmed a significant difference in medication adherence between patients with high and low emotional engagement. AF patients with greater emotional engagement reported better adherence to their medication regimens. Emotionally engaged patients are likely to perceive their medication regimen as a crucial component of their self-care,^64^ thus prioritising it even amid other life demands. Furthermore, they might be more inclined to seek support when facing challenges with medication adherence, whether derived from healthcare providers, family or support groups. Consistent with previous studies, high levels of emotional engagement often correlate with a stronger sense of responsibility and commitment to following prescribed treatments.^65^,^67^ This emotional bond to their health condition can drive patients to adhere more strictly to their medication schedules, understanding its importance in managing their symptoms and preventing complications such as stroke or heart failure.^9 66 67^ Interestingly, no significant difference in medication adherence was observed between patients with high and low cognitive-behavioural engagement. While patients may be knowledgeable and proactive about their health management, these attributes alone do not necessarily translate to better medication adherence. The lack of a significant difference suggests that cognitive engagement needs to be complemented by emotional support to improve adherence^12^ effectively. Even when AF patients understand the importance of their medication and are motivated to manage their health, emotional barriers such as anxiety, depression or a lack of perceived self-efficacy can hinder adherence.^68^ This highlights the need for healthcare providers to adopt a holistic approach that addresses both the emotional and the cognitive aspects of patient engagement.^69^

Our findings indicate a significant difference in the perception of quality of care among AF patients with high and low levels of engagement. In particular, AF patients with greater emotional and cognitive-behavioural engagement reported significantly better perceptions of the quality of care they received, indicating that individuals perceived their healthcare providers as more supportive of their autonomy and more motivated in their care management. The HCCQ assesses several critical aspects of patient-centred communication, including the degree to which healthcare professionals provide patients with options, express confidence in the patient’s ability to make health-related changes and seek to understand the patient’s perspective before making recommendations.^70^ Autonomy-supportive environments have enhanced patient engagement, where healthcare professionals provide clear and comprehensive information and encourage patient involvement in care decisions.^45^ Our findings corroborate the literature indicating that when patients perceive their healthcare providers as supportive and autonomy-enhancing, they are more likely to be engaged in their health management, adhere to their treatment and be more satisfied with their care process.^65 71^ This association has also been observed across various chronic conditions, where patient-centred communication is linked to better health outcomes and greater patient satisfaction.^72^ For AF patients, a supportive healthcare climate can lead to improved management of the condition, as these patients are more likely to adhere to complex treatment regimens and engage in necessary lifestyle changes.

Some limitations should be acknowledged when interpreting the findings of this study. The predominantly European sample, with a notable under-representation of participants from certain countries such as Denmark and Romania, may hinder the generalisability of the results to broader populations and cultural contexts. The cross-sectional design may limit the ability to establish causal relationships between patient engagement levels and health outcomes over time. Additionally, despite efforts to translate and validate the surveys, differences in language interpretation and cultural backgrounds may have influenced the consistency and comparability of responses. The present study did not include a control group of patients without AF but with similar comorbidities, which limits the ability to isolate the specific impact of AF on patient engagement and related outcomes. Future research should consider incorporating a matched control group to better understand how AF uniquely influences these factors. One limitation of this study is that differences between AF subtypes were not analysed. However, the management is similar across subtypes, with most patients receiving anticoagulants and antiarrhythmic drugs and presenting with multiple comorbidities. Nevertheless, it is recognised that symptom presentation and the psychological burden of the disease may vary between AF subtypes, potentially influencing how patients experience the condition. Finally, while patient-reported outcomes were comprehensively assessed, the study did not include direct clinical endpoints, which may have provided more robust insights into the direct impact of patient engagement on health outcomes. These limitations underscore the need for caution in generalising findings and highlight opportunities for future research to address these methodological considerations and expand the scope of inquiry in diverse patient populations.

## Conclusions

In conclusion, this study provides a nuanced understanding of patient engagement among individuals with AF and multimorbidity, highlighting the influence of various sociodemographic and health-related factors. Our findings underscore significant differences in engagement levels based on age, sex, educational attainment and a number of comorbidities, revealing distinct engagement personas within patients with AF and multiple morbidities. Emotional engagement was notably greater among older adults, reflecting their accumulated health management experience and emotional resilience. Conversely, younger adults demonstrated greater cognitive-behavioural engagement, indicative of proactive health management behaviours and greater health literacy. Importantly, our results also emphasise the positive associations between higher engagement levels and improved quality of life, medication adherence and perceptions of healthcare quality. These findings suggest that tailored interventions addressing both emotional and cognitive-behavioural aspects of engagement are essential for optimising health outcomes in AF patients with multimorbidity. Future research should further explore the longitudinal impact of engagement on clinical outcomes and refine strategies to enhance patient-centred care approaches across diverse healthcare settings.

## supplementary material

10.1136/bmjopen-2024-094351 online supplemental file 1

10.1136/bmjopen-2024-094351 online supplemental file 2

## Data Availability

Due to privacy and ethical restrictions, the datasets generated and/or analyzed during the current study are not publicly available, but they are available from the corresponding author upon reasonable request.
