# Supplementary material for: Exploring patient engagement in atrial fibrillation with multimorbidity: impact on quality of life, medication adherence and healthcare perceptions—a multicountry cross-sectional study
Source: BMJ Open. 2025 Mar 18;15(3):e094351. doi: 10.1136/bmjopen-2024-094351 (PMC11927486; doi:10.1136/bmjopen-2024-094351)
Supplement: online supplemental file 1 [file bmjopen-15-3-s001.docx]

**Appendix A.** Participants’ sociodemographic and health-related characteristics (*n* = 659)

| **Characteristics** | **Mean (SD), n (%)** |
| --- | --- |
| Age *(years)* | 70.9 (10.2) |
| Women | 348 (52.8) |
| White ethnicity‡ | 645 (97.9) |
| Country |  |
| UK | 358 (54.3) |
| Spain | 122 (18.5) |
| Romania | 92 (14.0) |
| Italy | 84 (12.7) |
| Denmark | 3 (0.5) |
| Educational attainment |  |
| Primary | 94 (14.7) |
| Secondary* | 266 (39.9) |
| Degree level or above | 280 (42.5) |
| Other/prefer not to say | 19 (2.9) |
| Number of comorbidities (*n* = 638) |  |
| ≤ 2 | 275 (41.7) |
| 3-5 | 297 (45.1) |
| > 5 | 66 (10.0) |

*IQR,* interquartile range*; SD,* standard deviation

‡6 (1.1%) identified as Hispanic or Latino ethnicity

*** High school and Apprentice/Professional Training/Vocational Training were included in the ‘Secondary level’ education group
