# Supplementary material for: Exploring patient engagement in atrial fibrillation with multimorbidity: impact on quality of life, medication adherence and healthcare perceptions—a multicountry cross-sectional study
Source: BMJ Open. 2025 Mar 18;15(3):e094351. doi: 10.1136/bmjopen-2024-094351 (PMC11927486; doi:10.1136/bmjopen-2024-094351)
Supplement: online supplemental file 2 [file bmjopen-15-3-s002.docx]

**Appendix B.** Pearson correlation coefficients among quality of life domains, medication adherence, perception of quality of healthcare, and patient engagement

| **Variables** | **1** | **2** | **3** | **4** | **5** | **6** | **7** | **8** | **9** | **10** |
| --- | --- | --- | --- | --- | --- | --- | --- | --- | --- | --- |
| 1. Mobility | - |  |  |  |  |  |  |  |  |  |
| 1. Self-care | 0.493^**^ | - |  |  |  |  |  |  |  |  |
| 1. Usual activities | 0.598^**^ | 0.505^**^ | - |  |  |  |  |  |  |  |
| 1. Pain/discomfort | 0.373^**^ | 0.274^**^ | 0.416^**^ | - |  |  |  |  |  |  |
| 1. Anxiety/depression | 0.184^**^ | 0.214^**^ | 0.229^**^ | 0.249^**^ | - |  |  |  |  |  |
| 1. VAS | -0.422^**^ | -0.377^**^ | -0.423^**^ | -0.421^**^ | -0.304^**^ | - |  |  |  |  |
| 1. MARS-5 | -0.116^*^ | -0.033 | -0.061 | -0.097^*^ | -0.058 | 0.155^**^ | - |  |  |  |
| 1. HCCQ | -0.117^*^ | 0.006 | -0.109^*^ | -0.192^**^ | -0.073 | 0.153^**^ | 0.226^**^ | - |  |  |
| 1. PHE-s | -0.258^**^ | -0.199^**^ | -0.299^**^ | -0.297^**^ | -0.437^**^ | 0.424^**^ | 0.111^*^ | 0.260^**^ | - |  |
| 1. ACE measure | -0.209^**^ | -0.236^**^ | -0.198^**^ | -0.122^*^ | -0.167^**^ | 0.203^**^ | 0.111^*^ | 0.245^**^ | - | - |

*ACE measure,* Altarum Consumer Engagement Measure; *HCCQ,* Health Care Climate Questionnaire; *MARS-5,* Medication Adherence Report Scale; *VAS,* visual analog scale; *PHE-s,* Patient Health Engagement Scale

**p* < 0.05, ***p* < 0.001
