# Supplementary material for: Exploring patient engagement in atrial fibrillation with multimorbidity: impact on quality of life, medication adherence and healthcare perceptions—a multicountry cross-sectional study
Source: BMJ Open. 2025 Mar 18;15(3):e094351. doi: 10.1136/bmjopen-2024-094351 (PMC11927486; doi:10.1136/bmjopen-2024-094351)
Supplement: Supplementary file 3 [file bmjopen-2024-094351.reviewer_comments.pdf]

PEER REVIEW HISTORY

BMJ Open publishes all reviews undertaken for accepted manuscripts. Reviewers are asked to complete a checklist review form and are provided with free text boxes to elaborate on their assessment. These free text comments are reproduced below.

ARTICLE DETAILS

Title (Provisional)

Exploring patient engagement in atrial fibrillation with multimorbidity: Impact on quality of life, medication adherence, and healthcare perceptions – A multicountry cross-sectional study

Authors

Bosio, Caterina; Usta, Dilara; Leo, Donato; Trevisan, Caterina.; Lane, Deirdre; GRAFFIGNA, GUENDALINA

VERSION 1 - REVIEW

**Reviewer**	**1**
**Name**	**Germanova, Olga**
**Affiliation**	**Samara State Medical University Library**
**Date**	**27-Nov-2024**
**COI**	**None**

Dear authors,

The topic of the article is interesting. The design, methods are adequate. However, there are several questions and recommendations.

1. Patients with atrial fibrillation of what types did you enroll in the study? The results can differ between the types.

2. Inclusion/Exclusion criteria?

3. There was no control group to compare the results who would undergo the same survey (maybe with the same comorbidities, but without AF).

4. When did you apply this survey? In admission to the hospital, when discharging from the hospital, in outpatient treatment? The results up to this can also be variable.

5. In the background, I recommend you to cite the following articles:

1) Germanova O, Galati G, Germanov A, Stefanidis A. Atrial fibrillation as a new independent risk factor for thromboembolic events: hemodynamics and vascular consequence of long

ventricular pauses. Minerva Cardiol Angiol. 2023 Apr;71(2):175-181. doi: 10.23736/S2724-5683.22.06000-8. Epub 2022 Mar 25. PMID: 35332747.

2) Germanova O, Koonts L, Reshetnikova Y, Sheifer M, Bikbaeva K, Kuvshinova N, De Berardis D, Galati G. Quality of Life Evaluation in Patients with Paroxysmal Atrial Fibrillation. Psychiatr Danub. 2024 Sep;36(Suppl 2):303-307. PMID: 39378487.

Reviewer **2**

Name **Kodani, Eitaro**

Affiliation **Nippon Medical School Tama Nagayama Hospital, Cardiovascular Medicine**

Date **23-Jan-2025**

COI **None**

This manuscript by Basio et al. focused on the association between patient engagement and quality performance indicators (QPIs) in patients with of atrial fibrillation (AF). Authors evaluated patient engagement using the Patient Health Engagement Scale (PHE-s) for emotional engagement and the Altarum Consumer Engagement Measure (ACE) for cognitive-behavioral engagement. Then, engagement scores of each measure were compared between 2 groups (low and high PHE-s or ACE). Authors demonstrated that the high emotional PE levels based on PHE-s were significantly more likely to be <75 years old, male, have a secondary level of education or above, and have <3 comorbidities. Regarding the ACE scores, the high cognitive-behavioral PE levels were more likely to be <65 years old, from Northern Europe. In addition, patients with high emotional PE demonstrated better quality of life, medication adherence, and perceptions of quality of care, whereas those with higher levels of cognitive-behavioral PE had better quality of life and perceptions of quality of care. As authors mentioned, although the importance of patient engagement in AF management has been recognized, it has not been established yet. Therefore, the concept of this study to explore it is valuable and results seem reasonable. Since this manuscript is written well, I do not have minor concern to be resolved. Authors may want to consider several minor issues as follows.

Minor comments

1) In abstract, once atrial fibrillation was abbreviated to AF, use it throughout the abstract.

2) Tables should be provided separately from the main text.

VERSION 1 - AUTHOR RESPONSE

Reviewer #1 Comments	Authors' response
Dear authors,<br>The topic of the article is interesting. The design, methods are adequate. However, there are several questions and recommendations.	
• Patients with atrial fibrillation of what types did you enroll in the study? The results can differ between the types.	We did not ask people to record their type of atrial fibrillation and, therefore, cannot report this information or examine differences between types of AF. However, the management is not so different between these types, i.e., patients are prescribed anticoagulants and, usually, antiarrhythmic drugs; moreover, in both cases, patients generally present with multiple comorbidities (more persistent ones) in addition to AF. What we can recognize is that the symptoms and psychological burden of the disease may slightly differ between AF types, so the experience of the disease by patients can change (please see https://doi.org/10.1093/europace/euv018). We added this to the limitations of the study (please see page 24).
• Inclusion/Exclusion criteria?	We have clarified this in the methods on page 6. *"Patients with AF were eligible for inclusion if they met the following criteria: (i) aged ≥ 18 years and (ii) the presence of at least one chronic comorbid condition. Exclusion criteria included: (i) inability to provide informed consent, (ii) moderate or severe cognitive impairment (e.g., dementia), (iii) inability to complete the survey online, (iv) the presence of health conditions that impede survey completion, and (v) unwillingness to participate."*
• There was no control group to compare the results who would undergo the same survey	There was no control group since the purpose of the survey was to assess patient engagement and related outcomes, specifically in individuals with

(maybe with the same comorbidities, but without AF).	AF. While we acknowledge the value of a control group without AF but with similar comorbidities, our study design aimed to capture engagement within the context of AF management. We have noted this as a study limitation in the manuscript. (please see page 23).
• When did you apply this survey? In admission to the hospital, when discharging from the hospital, in outpatient treatment? The results up to this can also be variable.	We have clarified this in the methods on page 6. *"Patients were invited to participate in the online survey through announcements on the Atrial Fibrillation Association (AFA) website or via healthcare professionals, including cardiologists, general practitioners, geriatricians, hematologists, and internal medicine specialists. These professionals were contacted via email through professional networks within the project consortium and invited to share the survey with patients attending clinical appointments at participating hospitals."*
• 5. In the background, I recommend you to cite the following articles:<br>1) Germanova O, Galati G, Germanov A, Stefanidis A. Atrial fibrillation as a new independent risk factor for thromboembolic events: hemodynamics and vascular consequence of long ventricular pauses. Minerva Cardiol Angiol. 2023 Apr;71(2):175-181. doi: 10.23736/S2724-5683.22.06000-8. Epub 2022 Mar 25. PMID: 35332747.<br>2) Germanova O, Koonts L, Reshetnikova Y, Sheifer M, Bikbaeva K, Kuvshinova N, De Berardis D, Galati G. Quality of Life Evaluation in Patients with Paroxysmal Atrial Fibrillation. Psychiatr Danub. 2024 Sep;36(Suppl 2):303-307. PMID: 39378487.	Thank you for suggesting additional references to support the Background. While we appreciate your recommendations, we feel they do not fully align with the focus of our manuscript and have therefore decided not to include them.
**Reviewer #2 Comments**	**Authors' response**

This manuscript by Basio et al. focused on the association between patient engagement and quality performance indicators (QPIs) in patients with of atrial fibrillation (AF). Authors evaluated patient engagement using the Patient Health Engagement Scale (PHE-s) for emotional engagement and the Altarum Consumer Engagement Measure (ACE) for cognitive-behavioral engagement. Then, engagement scores of each measure were compared between 2 groups (low and high PHE-s or ACE). Authors demonstrated that the high emotional PE levels based on PHE-s were significantly more likely to be <75 years old, male, have a secondary level of education or above, and have <3 comorbidities. Regarding the ACE scores, the high cognitive-behavioral PE levels were more likely to be <65 years old, from Northern Europe. In addition, patients with high emotional PE demonstrated better quality of life, medication adherence, and perceptions of quality of care, whereas those with higher levels of cognitive-behavioral PE had better quality of life and perceptions of quality of care. As authors mentioned, although the importance of patient engagement in AF management has been recognized, it has not been established yet. Therefore, the concept of this study to explore it is valuable and results seem reasonable. Since this manuscript is written well, I do not have minor concern to be resolved. Authors may want to consider several minor issues as follows.	
• Minor comments<br>1) In abstract, once atrial fibrillation was abbreviated to AF, use it throughout the abstract.	Thank you very much. We have revised the abstract.
• Tables should be provided separately from the main text.	We followed the general formatting guidelines across BMJ, which indicates the following: *"Tables should be in Word format and placed in the main text where the table is first cited"* (https://authors.bmj.com/writing-and-formatting/formatting-your-paper/)
